# ORGANIZATIONAL BARRIERS TO INCLUDING CAREGIVERS OF HOSPITALIZED PATIENTS LIVING WITH DEMENTIA

**DOI:** 10.1093/geroni/igae098.2230

**Published:** 2024-12-31

**Authors:** Sydney Hoel, Catherine Still, Teresa Thuemling, Andrea Strayer, Nicole Werner, Beth Fields

**Affiliations:** Indiana University, Bloomington, Bloomington, Indiana, United States; University of Wisconsin Madison, Madison, Wisconsin, United States; Indiana University Bloomington, Bloomington, Indiana, United States; University of Iowa, Iowa City, Iowa, United States; Indiana University, Bloomington, Bloomington, Indiana, United States; University of Wisconsin Madison, Madison, Wisconsin, United States

## Abstract

Caregivers play a critical role in managing the health and well-being of hospitalized family members or friends living with Alzheimer’s disease and related dementias. Yet, caregivers often report feeling excluded in hospital care. In order to effectively include caregivers in hospital care, the organizational barriers to inclusion faced by clinicians must be addressed by healthcare systems. The objective of this study was to identify organizational barriers to including caregivers of hospitalized patients living with dementia. We conducted semi-structured interviews with clinicians (n=26) who provide care to patients living with dementia in the hospital setting. Participants were nurses (42%), doctors (19%), social workers (12%), and rehab professionals (19%), and had an average of 11 years of experience in their role. Interview data were analyzed using a team-based content analysis directed by the Systems Engineering Initiative for Patient Safety (SEIPS) framework. We identified 6 categories of organizational barriers: culture, staffing, communication, policies, collaboration, and teamwork. Specifically, clinicians noted that organizations tend to emphasize “quantity over quality” culture, de-incentivizing and reducing time for interaction with caregivers. Most clinicians perceived a lack of organization support to include caregivers and could not recall receiving training on how best to include caregivers. These findings indicate multiple opportunities for healthcare systems to better support and prepare clinicians to include caregivers in hospital care. Information gleaned from this study can be utilized to guide the development of a more inclusive hospital organization, consistent with Goal 2 of the 2022 National Strategy to Support Family Caregivers.

